# Regeneration of Escherichia coli from Minicells through Lateral Gene Transfer

**DOI:** 10.1128/JB.00630-17

**Published:** 2018-04-09

**Authors:** Hideki Kobayashi

**Affiliations:** a Japan Agency for Marine-Earth Science & Technology, Yokosuka, Japan; Michigan State University

**Keywords:** bacterial conjugation, Hfr, regeneration of bacteria, minicells

## Abstract

Recently, artificial life has been created with artificial materials and methods. Life can be created when genomic DNA molecules are integrated in liposomes containing biochemical reactions for biogenic needs. However, it is not yet known whether the integration of these parts will be able to occur in nature and constitute a living system. I planned to regenerate bacteria from biologically active liposomes by inserting genomic DNA using only natural materials and methods. Minicells of Escherichia coli, containing plasmids and activated SOS proteins, act as protocells. Four new E. coli strains were regenerated from minicells by inserting the genomes by using the system for conjugation between F^−^ and Hfr strains. Cells of the four regenerated strains showed the same genetic markers as the two genome donors. Pulse-field gel electrophoresis of their genomes showed admixing of those of both donors. In addition, the genomes of the four regenerated strains had chimeric genome of the two donors. These results show that synthesis of life can occur in nature without artificial arrangement.

**IMPORTANCE** What is the difference between inanimate objects and organisms? Organisms always have genomic DNA. When organisms lose their genomes, they can neither grow nor reproduce. As the result, organisms turn into inanimate objects without their genomes. In this study, I regenerated microbes from cells that had lost their genomes (cell corpses) by inserting another genome. All steps of regeneration used the natural behavior of microbes. The same regeneration of microbes could happen in nature. These primitive lives have plasticity, which accelerates evolution and provides various kinds of life in the world.

## INTRODUCTION

Synthesis of artificial life is an important approach to gain a better understanding of life (1, 2). Some researchers have reported the construction of artificial biological reactions in artificially synthesized liposomes with a view toward the development of artificial life. DNA replication inside liposomes was demonstrated by a PCR-based assay (3). In addition, nucleic acid and protein syntheses have been carried out in liposomes (4–6). Furthermore, artificial gene cascades were shown to work in liposomes (7). A complete bacterial genome has been synthesized chemically (8), and the chemically synthesized genome worked in bacterial cells (9). On the other hand, Dolly the sheep was created from a nucleus-less oocyte by mechanical injection of another adult nucleus in 1996 (10, 11). With this background, life can be created when the genome DNA, protein synthesis system, and energy production system are assembled in a liposome. Researchers prepared prelife materials from organisms but set up the situation for creation of life by artificial methods. It is tempting to ask whether new organisms can be born in nature when the same situations as for artificial creation of organisms occur in a natural environment. To address this question, I attempted the reconstruction of a living system by combining liposomes and genomic DNAs. A minicell is a part of a bacterial cell produced by some bacteria with mutations in the genomic DNA in genes involved in cell division (12–14). The minicell has a system for synthesizing proteins, its membrane contains the electron transport chain to obtain energy, and it shows the same life-related activities as living cells with the exception of the reactions related to genomic DNA (15–17). In this study, I inserted an E. coli genome into minicells by conjugative gene transfer and succeeded in regenerating them as live E. coli cells.

## RESULTS AND DISCUSSION

### Preparation of the genome recipients.

**FIG 1:**
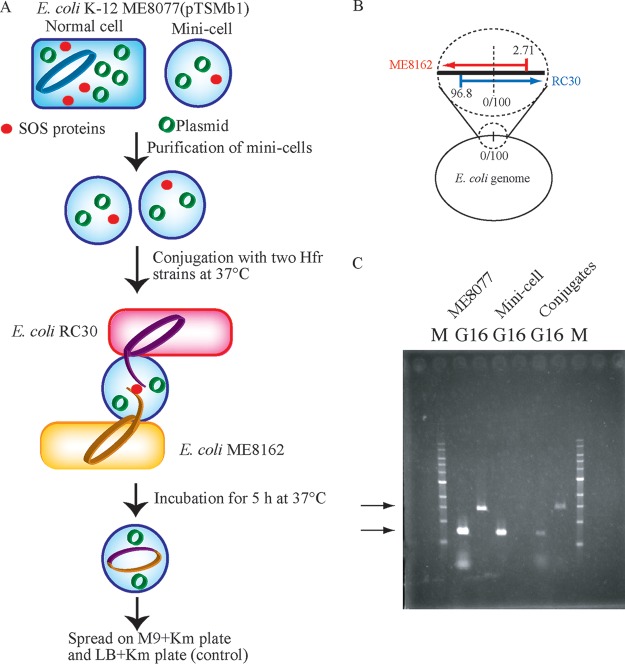
Strategy for regeneration of E. coli cells from minicells. (A) Plan for regeneration from purified minicells of E. coli ME8077 by insertion of the genomes of ME8162 and RC30 through conjugation with F^−^ and Hfr strains. (B) Point of origin and direction of insertion of the genomes of ME8162 and RC30 during conjugation. (C) PCR amplification of the GFP gene and the 16S rRNA gene of ME8162 carrying pTSMb1, purified minicells, and minicells after conjugation with Hfr strains. Lanes: G, GFP gene; 16, 16S rRNA gene. Arrows indicate amplified DNA fragments.

I used minicells of Escherichia coli ME8077 as genome recipients, because this allowed use of the conjugation between F^−^ and Hfr strains for insertion of genomic DNA (Fig. 1A) (18, 19). E. coli ME8077 was transformed with pTSMb1 to maintain the RNA polymerase concentration in the minicells (20). In addition, pTSMb1 remains in the minicells and can be used to distinguish the grown cells derived from minicells after conjugation with a *lac-cI* toggle switch (21). Minicells were purified by centrifugation and repeating filtration with appropriate filters within 30 min (22). In addition, ME8077 was incubated with a low concentration of mitomycin C, an antibiotic that attaches to DNA molecules, produces single-stranded DNA, and then activates the SOS signal cascade in E. coli. The genomic DNA inserted from the Hfr strain into the minicells would require repair because it is transferred in linear form, and the enzymes from the SOS cascade can promote DNA repair to construct the whole genome. In total, genome recipient liposomes were prepared from the minicells by the inclusion of pTSMb1, some SOS cascade proteins, and short single-stranded DNAs. The minicells were collected by centrifugation and suspended in 20 μl of M9 broth after filtration of 40 ml of ME8077 culture. I confirmed that no colonies grew when aliquots of 10 μl of the minicell suspension were spread on LB agar plates.

### Regeneration of E. coli cell.

**FIG 2:**
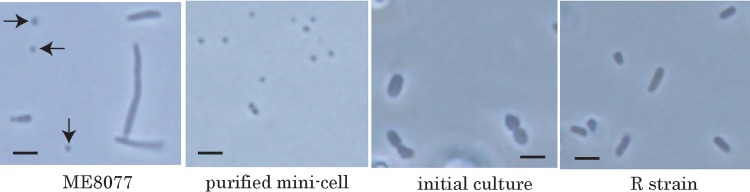
Cell shapes of ME8077, minicells, and regenerated cells. The minicells (arrows) were purified from ME8077 as described in Materials and Methods. All strains were incubated in LB medium at 37°C. Bars indicate 5 μm.

Two Hfr strains, ME8162 and RC30, were used as genome donors; these strains insert the genome counterclockwise from 2.71 min (HfrR5) and clockwise from 96.8 min (HfrH), respectively (Fig. 1B). Conjugation was carried out in M9 minimal medium with addition of 0.4% glucose as a carbon source for 5 h at 37°C to insert the whole genomes from the two Hfr strains. The presence of the E. coli genome and pTSMb1 was determined by PCR amplification of the 16S rRNA gene and green fluorescent protein (GFP) gene, respectively (Fig. 1C). ME8077 was positive for both genomic DNA and pTSMb1, and both the 16S rRNA gene and the GFP gene were detected by PCR. The purified minicells had only pTSMb1, and only the GFP gene was detected. PCR amplification also demonstrated that the minicells were successfully purified by filtration and contained pTSMb1. Both the 16S rRNA gene and the GFP gene were detected in the purified minicells after conjugation with ME8126 and RC30. These results demonstrated the successful insertion of genomic DNA by conjugation of minicells from the F^−^ and Hfr strains under these experimental conditions. The conjugates were grown on M9 agar plates containing 0.4% glucose and 100 μg/ml kanamycin (M9GK) at 37°C for 4 to 5 days. Four colonies grew on M9GK from over 10 experiments, and these four strains were designated R1 to R4. No colonies grew on M9GK agar plates when minicells were conjugated with only ME8162 or RC30. Figure 2 shows micrographs of ME8077, the purified minicell fraction, the first culture of the regenerated strain (R1), and the stable culture of the R1 strain. The minicells produced by ME8077 were spheres about 1 μm in diameter. At the initial phase, the four R strains had short rod-shaped cells (2 to 3 μm by 2 to 4 μm). However, the R strains were very unstable in morphology and grew very slowly. The cells were cultured for several generations to allow stabilization of their shapes and properties, and I then investigated whether they originated from minicells of ME8077 and had the genomes of ME8162 and/or RC30.

### Verification and characterization of the regenerated E. coli cells.

**FIG 3:**
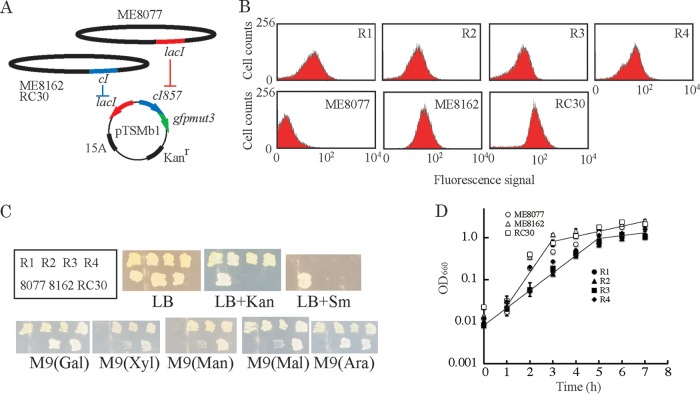
Properties of the regenerated cells. (A) The indicator plasmid, pTSMb1. The *cI* gene on the genomes of ME8162 and RC30 represses the expression of *lacI* of pTSMb1 and allows GFP gene expression. The *lacI* gene on the genome of ME8077 represses *cI* and GFP gene expression. (B) Expression of the GFP gene in regenerated cells and their parent strains. (C) Genetic markers of the regenerated cells and their parent strains. All strains were streaked on LB medium containing antibiotics or M9 medium containing each of various saccharides as the sole carbon source. (D) Growth of the regenerated cells and their parents. All strains were incubated aerobically (160 rpm) at 37°C, and growth was determined by measuring the optical density at 660 nm. Three experiments were carried out independently, and error bars indicate standard deviations (SD) (*n* = 3).

The *lac-cI* gene toggle switch on pTSMb1 shows bistable gene expression with the existence of the *lacI* and *cI* genes in genomic DNA (21). ME8077 has the *lacI* gene but not the *cI* gene, and it shows no GFP expression (off state). On the other hand, ME8162 and RC30 have *cI* but not *lacI*, and they show GFP expression (on state) (Fig. 3A and B). All regenerated cells from strains R1 to R4 showed GFP expression (on state) and carried the pTSMb1 plasmid (see Fig. S1 in the supplemental material). In addition, the R strains did not produce any minicells. These results suggested that the R strains were not spontaneous mutants of ME8077 that had lost the amino acid requirements. the R strains were also examined with regard to the genetic markers of ME8077, utilization of galactose, xylose, mannitol, maltose, and arabinose, amino acid requirements, and streptomycin resistance (Fig. 3C). R2, R3, and R4 showed the same genetic markers as RC30. R1 showed the same genetic markers as ME8162 with the exception of utilization of maltose. I attempted to obtain conjugational recombinants with genetic markers matching the R strains by conjugation of ME8077 with two Hfr strains under the same conditions. However, none of 243 conjugational recombinants grown on M9GK plates showed the same genetic markers, as well as GFP and minicell productivity, as the R strains (Table 1). In the case of a simple conjugation experiment, the possibility of multiple transfers of genetic markers between F^−^ and Hfr strains is less than 2% (23). Therefore, R strains cannot be obtained from the conjugational recombinants between ME8077 and ME8162 and/or RC30 from their physiological properties. The growth curves of the R strains were compared with those of their parent strains at 37°C (Fig. 3D). The doubling times of R1, R2, R3, and R4 were 0.59, 0.57, 0.57, and 0.45 h, respectively, which were in the normal range for E. coli at 37°C. The optical densities at 660 nm (OD_660_) of the R strains in the stationary phase were about 1.5, which was about one-third of those of ME8162, RC30, and ME8077, and the growth properties of the R strains were different from those of their parent strains.

**TABLE 1:** Analysis of unselected markers in both Thr^+^ and Leu^+^ recombinants from Hfr ME8162 and Hfr RC30 crosses with ME8077 as the recipient strain

No. of markers	Marker(s)^*a*^	% of recombinants^*b*^
1	*ara*	88.5
	*rpsL*	2.1
	*mtl*	21.8
	*mal*	31.7
	*xyl*	14.4
	*gal*	4.9
	*min*	1.2
	GFP gene	0.8
2	*ara*, *rpsL*	1.6
	*ara*, *mtl*	19.3
	*ara*, *mal*	28.4
	*ara*, *xyl*	12.4
	*ara*, *gal*	4.9
	*ara*, *min*	1.2
	*ara*, GFP gene	0.8
	*rpsL*, *mtl*	0.4
	*rpsL*, *mal*	0.8
	*rpsL*, *xyl*	0.8
	*mtl*, *mal*	11.1
	*mtl*, *xyl*	7.8
	*mtl*, *gal*	1.2
	*mtl*, *min*	0.4
	*mtl*, GFP gene	0.4
	*mal*, *xyl*	7.8
	*mal*, *gal*	0.8
	*mal*, GFP gene	0.4
	*xyl*, *gal*	2.1
	*gal*, *mal*	0.4
3	*ara*, *rpsL*, *mtl*	0.4
	*ara*, *rpsL*, *mal*	0.8
	*ara*, *rpsL*, *xyl*	0.4
	*ara*, *mtl*, *mal*	9.9
	*ara*, *mtl*, *xyl*	6.6
	*ara*, *mtl*, *gal*	1.2
	*ara*, *mtl*, *min*	0.4
	*ara*, *mtl*, GFP gene	0.4
	*ara*, *xyl*, *gal*	2.1
	*ara*, *gal*, *min*	0.4
	*rpsL*, *mtl*, *mal*	0.4
	*rpsL*, *mtl*, *xyl*	0.4
	*rpsL*, *mal*, *xyl*	0.4
	*mtl*, *mal*, *xyl*	6.1
	*mtl*, *mal*, *gal*	0.4
	*mtl*, *xyl*, *gal*	0.8
	*mal*, *xyl*, *gal*	0.4
4	*ara*, *mtl*, *mal*, *xyl*	4.9
	*ara*, *mtl*, *mal*, *gal*	0.4
	*ara*, *mtl*, *xyl*, *gal*	0.8
	*ara*, *mal*, *xyl*, *gal*	0.4
	*ara*, *rpsL*, *mtl*, *mal*	0.4
	*ara*, *rpsL*, *mtl*, *xyl*	0.4
	*ara*, *rpsL*, *mal*, *xyl*	0.4
	*rpsL*, *mtl*, *mal*, *xyl*	0.4
	*mtl*, *mal*, *xyl*, *gal*	0.4
5	*ara*, *mtl*, *mal*, *gal*, *xyl*	0.4
	*ara*, *rpsL*, *mtl*, *mal*, *xyl*	0.4

a Genetic markers (locations) are as follows: *ara* (1.4 min), *rpsL* (74.8 min), *mtl* (81.3 min), *mal* (91.4 min), *xyl* (80.3 min), *gal* (17.0 min), *min* (26.4 min), GFP gene (*att*λ [17.3 min] plus *lacI* [7.9 min]).

b The total number of recombinants analyzed was 243. Recombinants were analyzed by replica plating of colonies picked with a toothpick and regrown on selective agar. None of the recombinants showed other combinations of genetic markers or exchange of over six markers.

### Comparison of restriction fragments of the genomes.

**FIG 4:**
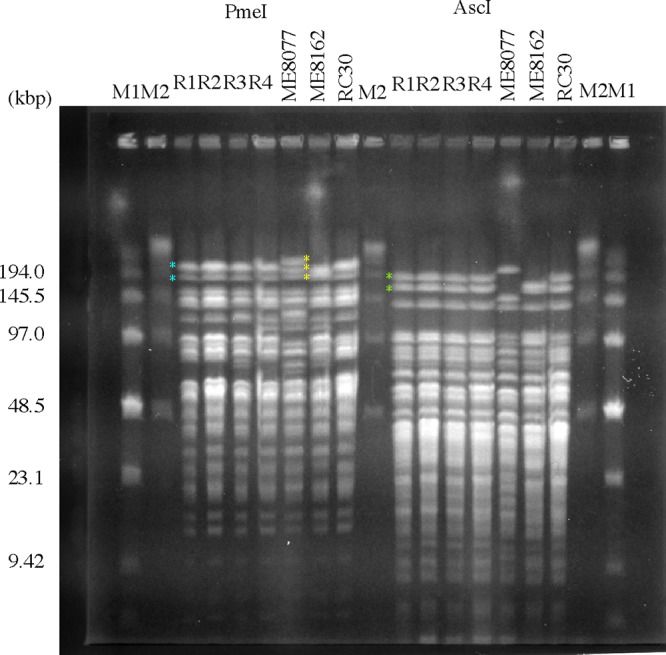
Pulsed-field gel electrophoresis of PmeI and AscI digests of the E. coli genome. Electrophoresis was carried out as described in Materials and Methods. M1 and M2 are the low-range PFG marker and lambda ladder marker, respectively. Asterisks show bands of interest, as described in the text.

The genomes of the R strains, ME8077, ME8162, and RC30 were also investigated to identify their origin. Restriction analyses of the genomes were performed using PmeI or AscI, and the resulting fragments were separated by pulsed-field gel electrophoresis (PFGE) (Fig. 4). The digestion patterns of the ME8077, ME8162, and RC30 genomes with PmeI and AscI were quite different from each other. For example, there were three bands of over 194 kb in the ME8077 genome (yellow asterisks) digested with PmeI, whereas both ME8162 and RC30 showed only two bands (blue asterisks). The differences in digest patterns with AscI between ME8077, ME8162, and RC30 were clearer. The digestion pattern of ME8077 was markedly different from those of the other two Hfr strains between 9.42 kb and 200 kb. The PmeI and AscI digest patterns of the genomic DNAs of the R strains were similar to those of a mixture of ME8162 and RC30 and dissimilar to those of ME8077. There were some differences in the genomic DNA digest patterns among the R strains. All R strains showed the same pattern as RC30 from 220 to 125 kb on digestion with PmeI. However, R3 and R4 showed the same pattern as ME8162 from 97 to 70 kb and from 125 to 80 kb, respectively, on digestion with PmeI. R1 and R2 showed the same digest pattern as RC30 in this region. On the other hand, the genomes of all R strains showed the same pattern on digestion with AscI. The largest two fragments, of 190 and 165 kb (green asterisks), were the same as those of RC30. The digest patterns of the R strains from 105 to 93 kb were the same as those of ME8162, and those from 93 to 72 kb and from 23 to 7.7 kb were the same as those of RC30. The genomes of the four R strains were closer to that of RC30 than to that of ME8162 based on the results of PFGE. The genome of the ME8162 strain carried a lambda lysogen. The minicells into which mainly the genome of ME8162 had been inserted would lyse by the lambda phage lytic pathway mediated by activation of RecA protein on stimulation with mitomycin C (24). These results clearly indicated that the genomes of the R strains were constructed from those of both ME8162 and RC30. The R strains were not spontaneous mutants or recombinants of pTSMb1 of ME8162 or RC30.

### Genome sequencing of E. coli strains.

**FIG 5:**
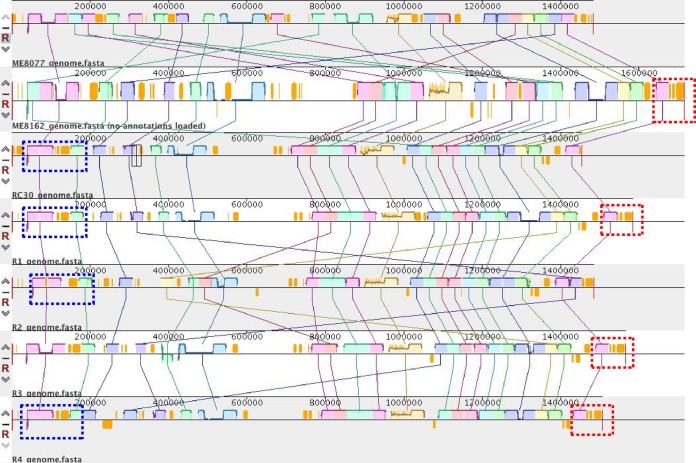
Comparative analyses of the E. coli genomes. Homologous regions are shown in boxes with the same colors. I used Mauve software for comparative analysis (locally colinear block weight = 22,195). Blue or red dashed boxes indicate regions homologous to RC30 or ME8162, respectively.

I also carried out genome sequencing of ME8077, ME8162, RC30, and R1 to R4 to characterize the genomes of the R strains (Table 2). The total contig length of ME8077 was about 4.34 Mb, which was smaller than those of other E. coli strains. The *cI* gene, which turns the toggle switch to the on state, was found in the contigs of R strains (R1, GenBank accession number BFAM01000002; R2, BFAN01000008; R3, BFAO01000001; R4, BFAP01000003). I arranged the contigs of each strain through homology searching with the W3110 genome data (GenBank accession number AP009048.1) (see Fig. S2 in the supplemental material). I then carried out a comparative analysis of strains R1 to R4 with the genome donor and recipient stains (Fig. 5). The genomes of the R strains contained regions similar to those of RC30 (blue dashed boxes) and ME8162 (red dashed boxes). The genome of R1 was especially close to that of RC30, other than the regions indicated by the red dashed boxes. The genomes of the R strains were different from each other. The genome of ME8077 was also different from those of the ME8162, RC30, and R strains.

**TABLE 2:** Summary of genome sequences of E. coli strains

Strain	No. of contigs	Total contig length (bases)	Avg contig length (bases)	N50^*a*^ (bases)	GC content
ME8077	77	4,342,381	54,279	147,478	0.5085
RC30	70	4,605,024	64,859	176,854	0.5071
ME8162	87	4,711,433	54,154	113,329	0.5068
R1	82	4,607,925	56,194	133,026	0.5071
R2	77	4,606,274	61,416	173,740	0.5071
R3	79	4,588,470	57,355	133,026	0.5072
R4	73	4,636,261	63,510	176,498	0.5072

a N50, shortest sequence length at 50% of the genome.

Therefore, I concluded that the R strains were new artificial E. coli strains derived from minicells isolated from ME8077 with a new genome comprised of a composite of those of ME8162 and RC30. There were consensus regions in ME8162 and MC30, and I could not find critical points of inserted genomes between the two genome donors. Furthermore, the genomes of the R strains had rearrangements or replacements, which would be due to homologous recombination and SOS system activity during conjugation. More genome sequencing will be needed for detailed analysis.

Other minicells, of the 1411 (CGSC6397) strain, were also examined; however, no colonies grew by conjugation with ME8162 and RC30. Seven other Hfr strains (see Table S1 in the supplemental material) in which the location and direction of Hfr were different from those of ME8162 and RC30 were also used as genome donor. However, regeneration of E. coli from minicells was unsuccessful using Hfr strain pairs other than ME8162 and RC30. I also could not obtain progeny in the case of conjugation with three or more Hfr strains, including ME8162 and RC30. The frequency of genome insertion from Hfr to F^−^ strain, during conjugation is dependent on the properties of each Hfr strain (18). Furthermore, the problem of lethal zygosis arises from the increase in Hfr cells during the long conjugation time of 5 h in this experiment (25). The conjugation conditions would affect regeneration of E. coli cells from minicells.

The strategy used in this study is similar to the nuclear transfer cloning technology applied to mammalian cells (10), with the minicells being analogous to oocytes enucleated by mechanical manipulation. However, there are two differences between this method and cloning technology. The presented method applied only natural processes and produced new organisms with a new genome rather than clones. The same events may occur in the natural habitat of E. coli, which is the lower intestines of warm-blooded animals, where more then hundreds of trillions of microorganisms are struggling for existence (26). As the method described here makes use of the unique conjugation system of E. coli for genome insertion, other methods to insert the genomic DNA would be needed for the construction of other forms of artificial life based on other species. Many proteins from other bacterial species have been expressed in minicells, and a foreign genome could be activated by expression of a suitable RNA polymerase, DNA polymerase, and ribosomes (27–29). Moreover, the results of the present study suggest that life can be derived from biologically active liposomes and a suitable genome.

## MATERIALS AND METHODS

### Strains, plasmids, growth conditions, and chemicals.

E. coli ME8077 (*minA minB thr-1 leuB6 azi lacY1 tsx ara-13 gal-6 malA1 thi-1 xyl-7 rpsL mtl-2 tonA2 supE44*) was used as the minicell producer (12). E. coli ME8162 (*lac gal xyl mtl malA thi* λ^+^) and E. coli RC30 (CGSC6599) (*lacZ* ΔM15 *lacI22* e14^−^ *relA1 spoT1 thi-1*) were used as genome donors and had HfrR5 (*leuA* → *tonA*) and HfrH (*valS* ← *attP4*), respectively (30, 31). The indicator plasmid, pTSMb1, had the *lac-cI* toggle switch, p15 origin of replication, and kanamycin resistance gene (21). E. coli strains were grown aerobically in LB medium (Difco) at 37°C unless otherwise indicated. M9 minimal medium was used for conjugation of minicells and Hfr strains as well as for confirmation of genetic markers of E. coli. Kanamycin and saccharides were purchased from Wako Chemical, Inc. Mitomycin C was purchased from Kyowa Hakko Kogyo Co., Ltd.

### Isolation and purification of minicells.

E. coli ME8077 was transformed with pTSMb1 and incubated in 40 ml of LB liquid medium containing 100 μg/ml of kanamycin and 20 ng/ml of mitomycin C at 37°C for 12 h. Cultures were filtered with membrane filters (Millex-SV [Millipore]; pore size, 5.0 μm). The filtrate was also refiltered twice with membrane filters (Millex-AA [Millipore]; pore size, 0.8 μm) to remove normal cells. Minicells were harvested by centrifugation of the filtrate (10,000 rpm, 5 min, 4°C), washed with M9 medium, and suspended in 20 μl of M9 medium. I spread 10 μl of this minicell fraction on LB agar plates to check for contamination by normal cells based on the growth of colonies.

### Conjugation conditions.

ME8162 and RC30 were incubated aerobically in LB liquid medium at 37°C. When the optical density at 660 nm (OD_660_) of cultures reached 0.1, cells were harvested by centrifugation (8,000 rpm, 1 min, 4°C), washed with the same volume of M9 medium, and then suspended in the same volume of M9 medium. Aliquots of 5 μl of the minicell fraction and 1 μl of each cell suspension were mixed and incubated for 5 h at 37°C. The mixture was then added to 100 μl of M9 medium and stirred with a vortex mixer for 3 to 5 min. The mixture was spread on M9 agar plates and incubated at 37°C.

### PCR amplification of the 16S rRNA gene and GFP gene.

PCR amplification was carried out with a Gene Amp PCR system 9700 (Applied Biosystems) using EX *Taq* polymerase (TaKaRa). The primers 27F (AGAGTTTGATCCTGGCTCAG) and 1492R (GGTTACCTTGTTACGACTT) were used for amplification of the 16S rRNA gene. The primers gfp-f (ATGAGTAAAGGAGAAGAACTTTT) and gfp-r (TTATTTGTATAGTTCATCCATGC) were used for amplification of the green fluorescent protein (GFP) gene.

### Assay of GFP expression.

Cells were collected by centrifugation (8,000 rpm, 1 min, 4°C), washed with phosphate-buffered saline (PBS) (75 mM sodium phosphate and 67 mM NaCl, pH 7.4), and suspended in PBS to measure GFP expression. All GFP expression data were collected using a Becton Dickinson FACSCalibur flow cytometer. Each fluorescence measurement of gene expression was obtained from 30,000 cells.

### PFGE of E. coli chromosomal DNA.

E. coli chromosomal DNA was prepared by the method of Heath et al. (32). E. coli strains were grown in 1 ml of LB medium for 12 h at 37°C. Cells were collected by centrifugation (8,000 rpm, 1 min, 4°C), washed with an equal volume of PIV buffer (1 M NaCl, 10 mM Tris-HCl, pH 7.6), and suspended in a half volume of PIV buffer. The cell suspension was mixed with an equal volume of preheated (50°C) 1.6% pulsed-field-certified (PFC) agarose (Bio-Rad) solution and poured immediately into a plug mold (Bio-Rad). The plug mold was kept at 4°C to allow the agarose to harden. An agarose gel block was removed from the plug mold and soaked in 1 ml of EC buffer (6 mM Tris-HCl, pH 7.6, 1 M NaCl, 100 mM EDTA, 0.5% polyoxyethylene acetyl ether [Brij 58], 0.2% deoxycholate, 0.5% *N*-lauroylsarcosine sodium). The agarose block in EC solution was then shaken gently for 15 min at room temperature. The agarose block was moved into 1 ml of EC buffer containing 1 mg/ml of lysozyme and 20 μg/ml of RNase and shaken gently overnight at 37°C. The agarose block was then transferred into 1 ml of ES buffer (0.5 M EDTA [pH 9.0], 0.5% *N*-lauroylsarcosine sodium) and shaken gently for 15 min at room temperature. The agarose block was transferred into 1.5 ml of ES buffer containing 50 μg/ml of proteinase K (Wako) and incubated for 36 h at 50°C. The agarose gel block was washed twice with 1.5 ml of Tris-EDTA (TE) buffer, transferred into 1.5 ml of TE buffer containing 1 mM phenylmethylsulfonyl fluoride (PMSF), and incubated for 1 h at room temperature. The agarose gel block was washed with TE buffer twice and kept as the chromosomal DNA gel block in TE buffer at 4°C. The agarose gel block was washed with the appropriate buffer for the restriction enzyme used (AscI or PmeI). The chromosomal DNA in the agarose gel block was digested with 30 U of AscI or PmeI for 24 h at 37°C and washed with TE buffer twice after the enzyme reaction. The agarose gel block was loaded into the well of a 1% PFC agarose gel for pulsed-field gel electrophoresis (PFGE) using a CHEF Mapper system (Bio-Rad) in 0.5× Tris-Borate-EDTA (TBE) buffer at 14°C in accordance with the manufacturer's instructions. For separation of fragments from 200 to 10 kb, the parameters were set to a pulse time of 0.47 to 17.33 s, a ramping factor of −1.357, a voltage of 6 V cm^−1^, and a run time of 20.3 h. DNA size markers, i.e., lambda ladder (727.5 to 48.5 kb) and low-range PFG marker (194 to 2.03 kb), were purchased from New England BioLabs.

### Genome sequencing of E. coli strains.

The E. coli genome was extracted by using the Gram-negative bacterial protocol of the DNeasy blood and tissue kit (Qiagen, Venlo, The Netherlands) from a culture grown in 10 ml of LB for 12 h at 37°C. One hundred nanograms of genomic DNA was sheared into about 400 to 800 bp with Covaris S220 (Covaris Inc., MA). After ligation with the barcode nucleotide, I prepared the DNA libraries for sequence with Kapa Hyper Prep kit (Kapa Biosystems, Wilmington, MA) according to manufacturer's protocol. The sequence library DNA was analyzed using MiSeq (Illumina Inc., San Diego, CA). By using CLC Genomics Workbench 9.0.1, I trimmed the barcode sequence. Subsequently, I performed a *de novo* assembly of DNA sequence data. The resulting sequences of contigs were used for subsequent analysis. In comparative analysis of each genome sequences, I used Mauve software from the Darling lab (http://darlinglab.org/mauve/user-guide/introduction.html).

### Accession number(s).

The GenBank accession numbers of the E. coli contigs are as follows: ME8077, BFAJ01000001 to BFAJ01000077 (77 entries); RC30, BFAL01000001 to BFAL01000070 (70 entries); ME8162, BFAK01000001 to BFAK01000087 (87 entries); R1, BFAM01000001 to BFAM01000082 (82 entries); R2, BFAN01000001 to BFAN01000077 (77 entries); R3, BFAO01000001 to BFAO01000079 (79 entries); and R4, BFAO01000001 to BFAO01000079 (79 entries).

## Supplementary Material

Supplemental material
