# Supplementary material for: Regeneration of Escherichia coli from Minicells through Lateral Gene Transfer
Source: J Bacteriol. 2018 Apr 9;200(9):e00630-17. doi: 10.1128/JB.00630-17 (PMC5892112; doi:10.1128/JB.00630-17)
Supplement: Supplemental material [file supp_200_9_e00630-17__index.html]

Supplemental material 

# Regeneration of Escherichia coli from Minicells through Lateral Gene Transfer

## Supplemental material

- Supplemental file 1 -

  Fig. S1 (pTSMb1 and plasmids of regenerated cells) and S2 (Arrangement of contigs based on homology to W3110 genome), Table S1 (*E. coli* Hfr strains used as genome donor), and supplemental text

  PDF, 3.5M
