# Supplementary material for: Regeneration of Escherichia coli from Minicells through Lateral Gene Transfer
Source: J Bacteriol. 2018 Apr 9;200(9):e00630-17. doi: 10.1128/JB.00630-17 (PMC5892112; doi:10.1128/JB.00630-17)
Supplement: Supplemental material [file JB.00630-17_zjb999094717s1.pdf]

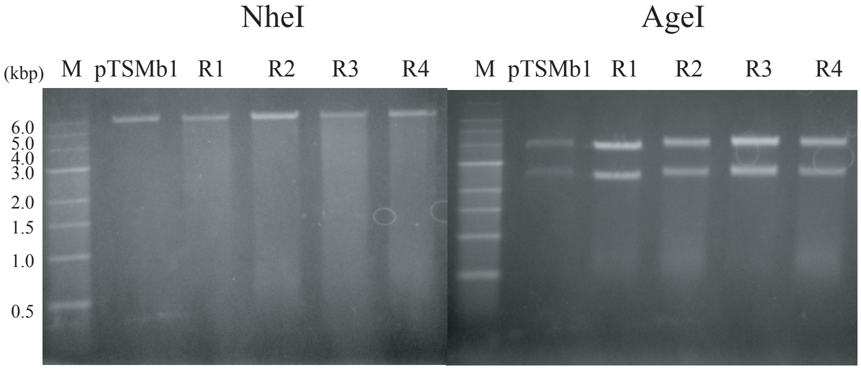

Figure S1 pTSMb1 and the plasmids of regenerated cells. The plasmids were isolated from 6 ml of overnight cultures using a mini-prep kit (Qiagen). The plasmids were digested with NheI or AgeI and separated by electrophoresis.

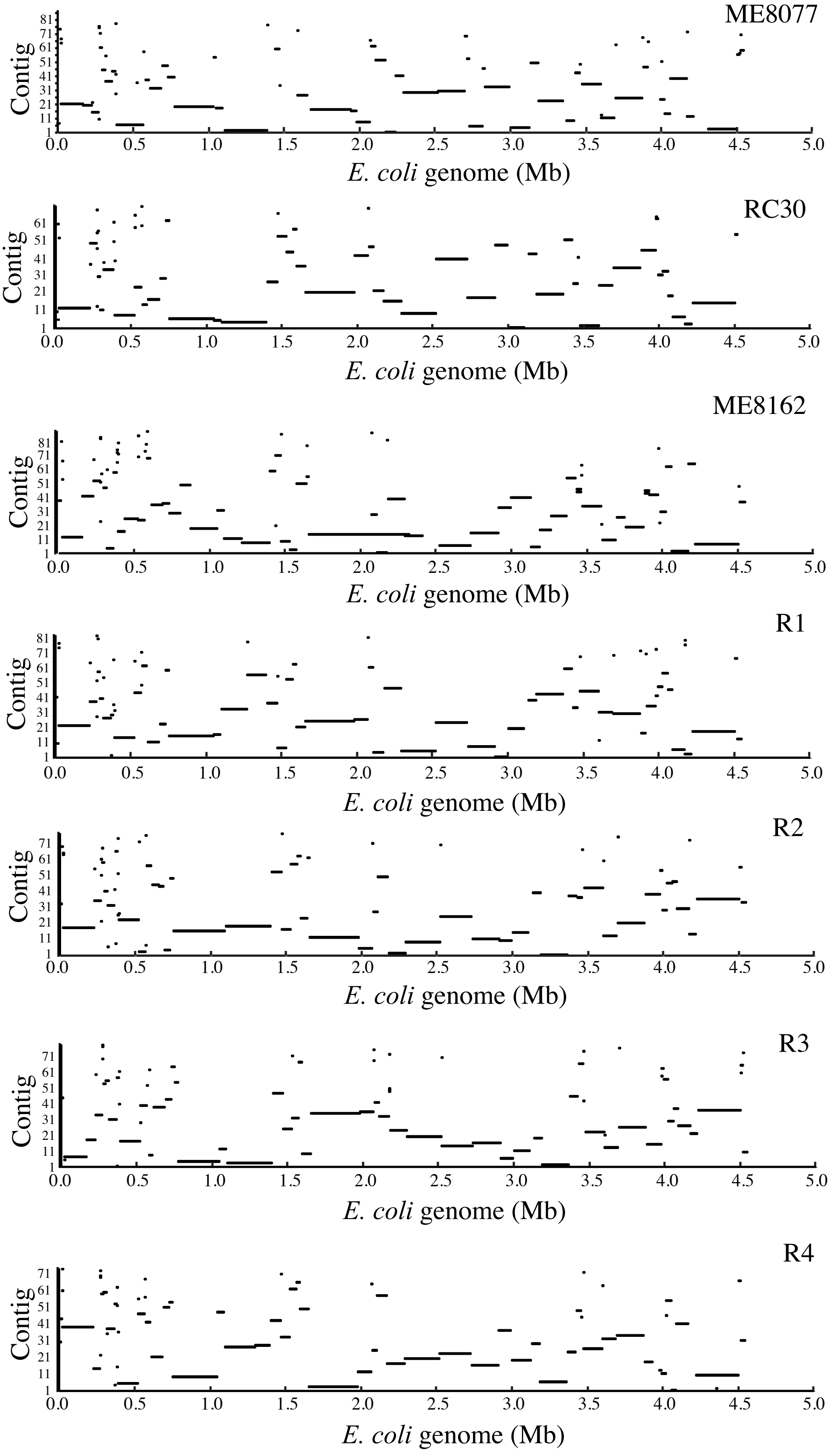

Figure S2 Arrangement of contigs based on homology to W3110 genome.

Each bar shows a contig and its position in W3110 genome.

Supplementary discussion

Unsuccessful experiments

I started conjugation of 9 Hfr strains (ME8162, RC30, and Table S1) and 2 mini-cell producers. I purified mini-cells from the cultures of ME8077 and CGSC6397 carrying pTSMb1 with/without addition of mitomycin C. The minimum inhibitory concentration of mitomycin C was 625 ng/ml in both mini-cell producers. I determined mitomycin C concentration to 20 ng/ml from mini-cell productivity under microscopic observation. I didn't measure the concentration of mini-cells during experiment, to use the mini-cells for conjugation as soon as possible. The conjugation experiment of mini-cell and Hfr strains was carried out while changing Hfr strains. When multiple Hfr strains were used for conjugation, I adjusted OD_{660} of each culture to 0.1, and used up to 3 kinds of Hfr strains in one experiment. The conjugation time was tested 1 h, 3 h, 5 h, and over night. I tried three times of experiments, and obtained 1 colony (R1) at 5 h conjugation with ME8162 and RC30 strains to the mini-cells isolated from mitomycin C added culture. Then, I repeated

conjugation experiments at the same condition to obtain more colonies, and two colonies (R2, R3) and one colony (R4) during 12 experiments. I also tried one or two other Hfr strains in addition to ME8162 and RC30 in conjugation experiment; however, I cannot obtain any colony. To increase efficiency of regeneration, I changed ME8162, RC30, and mini-cells concentration; however, I cannot obtain regenerated cells.

Supplemental Table 1 *E. coli* Hfr strains used as genome donor

Strain	Hfr position[1] (min)	Markers
CGSC#312	*tol*C -<-*rpo*D (68.8)	Hfr(PO12), *thr-1*, *leuB6*(Am), *lacZ4*, *glnX44*(AS), λ^-, *rfbC1*, *rpsL8*, *thiE1*
CGSC#2437	*arg*F->-*lac* (6.8)	Hfr(PO3), *glnX44*(AS)*?*, *hisG4*(Oc), *rpsL31*(strR), *mtl-1*, *thiE1*, *uvrA6*
CGSC#5132	*ksg*D->-*trg* (31.6)	Hfr(PO43), λ^-, *fabA2*(ts), *relA1?*, rpsL118(strR), malT1(λR), xyl-7, mtlA2, thiE1?

CGSC#4895	*leu*->-*fhu*A (77.4)	Hfr(PO111), *thr-1*, *leuB6*(Am), *fhuA2::IS2*, *lacY1*, *glnX44*(AS), *gal-3*, *malT1*(λR), *xyl-7*, *mtlA2*, *thiE1*
CGSC#5350	*his*->-*mgl* (46.7)	Hfr(PO44), *lacZ2210*(Am), *tsx-85*, *λ*-, *serU74*(ts,AS), *gyrA21*(NalR), *relA1*, *rpsL183*(strR), *spoT1*, *thiE1*
CGSC#5778	*lys*A->*ser*A (65.0)	Hfr(PO45), *lacZ105*(Am), *relA1*, *rpsL226*(strR), *thiE1*
CGSC#5816	lac-<-proC (8.6)	Hfr(PO129), *Sfa*(att)*7*, *thyA117*, *rpsL228*(strR), *metB1*, *creD101*

1. ->-; clockwise, -<-; counter clockwise
